# Effectiveness of iron supplementation in the perioperative management of total knee arthroplasty: a systematic review

**DOI:** 10.1186/s43019-020-00064-1

**Published:** 2020-08-28

**Authors:** Seung Hoon Lee, Joong Il Kim, Wonchul Choi, Tae Woo Kim, Yong Seuk Lee

**Affiliations:** 1 Department of Orthopaedic Surgery, Veterans Health Service Medical Center, Seoul, South Korea; 2 Department of Orthopaedic Surgery, Hallym University Kangnam Sacred Heart Hospital, Seoul, South Korea; 3 Department of Orthopaedic Surgery, CHA University, CHA Bundang Medical Center, Seongnam-si, South Korea; 4 Department of Orthopaedic Surgery, Seoul Metropolitan Government-Seoul National University Boramae Medical Center, Seoul, South Korea; 5 Department of Orthopaedic Surgery, Seoul National University College of Medicine, Bundang Hospital, Seoul, South Korea

**Keywords:** Total knee arthroplasty, Transfusion, Iron supplement, Hemoglobin, Iron

## Abstract

**Introduction/purpose:**

The purpose of this systematic review was to evaluate the effect of iron supplementation during total knee arthroplasty (TKA): (1) Is the iron supplementation necessary during TKA? (2) When is the optimal timing of iron supplementation? (3) Which is better, between orally and intravenously administered iron supplementation? And (4) What is the optimal dose of iron supplementation?

**Materials and methods:**

A rigorous and systematic approach was used and each of the selected studies was evaluated for methodological quality. Data about study design, total number of cases enrolled, iron administration method, timing, and dose were extracted. Change in hemoglobin and transfusion rates were extracted to evaluate the effectiveness of iron supplementation.

**Results:**

Eleven studies were included in the final analysis. Most of studies reported that hemoglobin change between iron and control group did not show any difference. Only one study reported that iron supplementation could reduce the decrease in hemoglobin. However, transfusion rate showed a decrease in the iron supplementation group compared with the control group. There was no clear consensus on the optimum timing and dose of iron supplementation and intravenously administered iron was more effective than orally administered iron, especially in anemic patients.

**Conclusion:**

Iron supplementation is not clear as a way to raise hemoglobin levels after TKA, but an effective treatment for lowering transfusion rate, especially in patients with anemia. We could not determine the optimal timing and dose of the iron. Intravenously administered iron was similar to, or better than, orally administered iron for improving hemoglobin levels and transfusion rate.

## Introduction

Total joint arthroplasty (TJA) is a successful and cost-effective treatment option for end-stage osteoarthritis (OA) [1]. However, TJA is an invasive procedure that can cause serious complications, such as blood loss, which is a major concern. Despite numerous studies on blood loss and its management during TJA, but there is little consensus about the amount of blood loss and management strategies for reducing the blood loss. This gap in our knowledge could be the differences in characteristics of the patient groups, surgical method, or the method used for the estimation of blood loss. Although there may be differences in blood loss, the decrease of hemoglobin after TKA is about 3 g/dL [2]. This often leads to allogenic transfusion, from 3% up to 69% [3, 4]. THA is associated with a higher transfusion rate than TKA [5]. THA is usually performed to treat hip fracture, whereas TKA is performed as a regular operation. There are more opportunities to improve the hemoglobin levels in the case of TKA, and the epidemiology of the patients who undergo TKA is also different from that of patients undergoing THA.

Allogenic transfusion can lead to increased length of stay, perioperative infection, and increased cost of treatment. Many methods have been tried to reduce allogenic transfusion, such as oral and intravenous administration of tranexamic acid, or intra-articular or subcutaneous routes to reduce transfusion rate [6–9]. The auto-transfusion system is a method that reduces allogenic transfusion during TKA [10]. Erythropoietin is also used to reduce the transfusion rate without adverse effects [11]. However, it is impossible to predict the exact effect of these methods, as they have been selected according to the needs of each patient, under different conditions [12–15].

Iron supplementation is widely accepted as an effective method for treatment of anemia during surgery. Iron can be administered orally or intravenously and is also available in high and low doses as per the requirement. There is little consensus regarding the method and optimal dose of iron supplementation. Iron supplementation can be used easily with few adverse effects, and it is also cost-effective [16]. Therefore, it can be a useful tool for perioperative blood management of TKA. Some meta-analyses have also reported the use of iron supplementation during various surgeries [17–19]. As blood loss can be different for each operation or surgical method, it is difficult to formulate a consistent conclusion after analyzing these heterogeneous surgical methods using pooled analysis. Instead, analysis should be performed individually for each surgical method. We believe that a separate analysis of the perioperative blood management in TKA is necessary.

The purpose of this study was to analyze the perioperative blood management in TKA. We included studies that compared the effect of iron supplementation under the conditions similar to those seen in TKA. We also wanted to evaluate the effect of iron supplementation in the perioperative blood management of TKA. Our systematic review was conducted to answer the following questions by analyzing studies that assessed iron supplementation for the perioperative blood management of TKA: (1) Is the iron supplementation necessary during TKA? (2) When is the optimal timing of iron supplementation? (3) Which is better, between orally and intravenously administered iron supplementation? And (4) What is the optimal dose of iron supplementation?

## Material and methods

### Search strategy

Rigorous and systematic approach conforming to the Preferred Reporting Items for Systematic review and Meta-Analysis (PRISMA) guidelines was used to verify our research question [20]. In phase 1 of the PRISMA search process, selected databases, including MEDLINE, EMBASE, and Cochrane database were searched (31 March 2020). A Boolean strategy was used, and all field search terms included the following: Search ([lower limb arthroplasty] OR [total knee arthroplasty]) AND ([iron] OR [ferric] OR [ferrous]). The references in the included studies were also screened, and unpolished articles were also checked manually. The bibliographies of the relevant articles were subsequently cross-checked for articles not identified in the search. In phase 2, abstracts and titles were screened for relevance. In phase 3, the full text of the selected studies was reviewed according to the inclusion criteria, and methodological appropriateness was determined with a predetermined question. In phase 4, the studies were subjected to an appropriate systematic review process.

### Eligible criteria

The following eligibility criteria were used to select studies: (1) the articles should be about TKA, (2) the articles should be written in English, (3) the full text of the article should be available, (4) the articles should include human in-vivo studies, and (5) the articles should include comparative studies about the use of iron use. The exclusion criteria were as follows: (1) articles not related to TKA, (2) articles including studies about perioperative blood management with selective iron use, (3) articles published before 2000, and (4) articles that were not clinical studies (experimental or review article).

### Data assessment

Each of the selected studies was evaluated by two independent authors for methodological quality. Data were extracted by the following standardized protocol: publication year, publication journal, study type, number of cases, type of iron supplement, timing, and dose of iron supplement, change in the levels of hemoglobin, serum ferritin and transferrin saturation, and transfusion rate. The extracted data were then cross-checked for accuracy and any disagreement were settled by the third contributing author. To assess the methodological quality of the studies, quality assessment was conducted using the Modified Coleman Criteria (Appendix A) [21]. The Modified Coleman Criteria has a scaled potential score ranging from 0 to 100. Scores of 85–100 are excellent, 70–84 are good, 55–69 are fair, and scores < 55 are poor. It is used for assessing the quality of surgical studies. Part A includes study size, mean follow-up, type of study, and diagnostic certainty. Part B includes outcome criteria, procedure for assessing clinical outcomes, description of patient selection process, post-operative rehabilitation, surgical technique, complications recorded, and revisions recorded. These criteria were created by two authors to quantify the quality of studies.

## Results

### Search

**Fig. 1:**
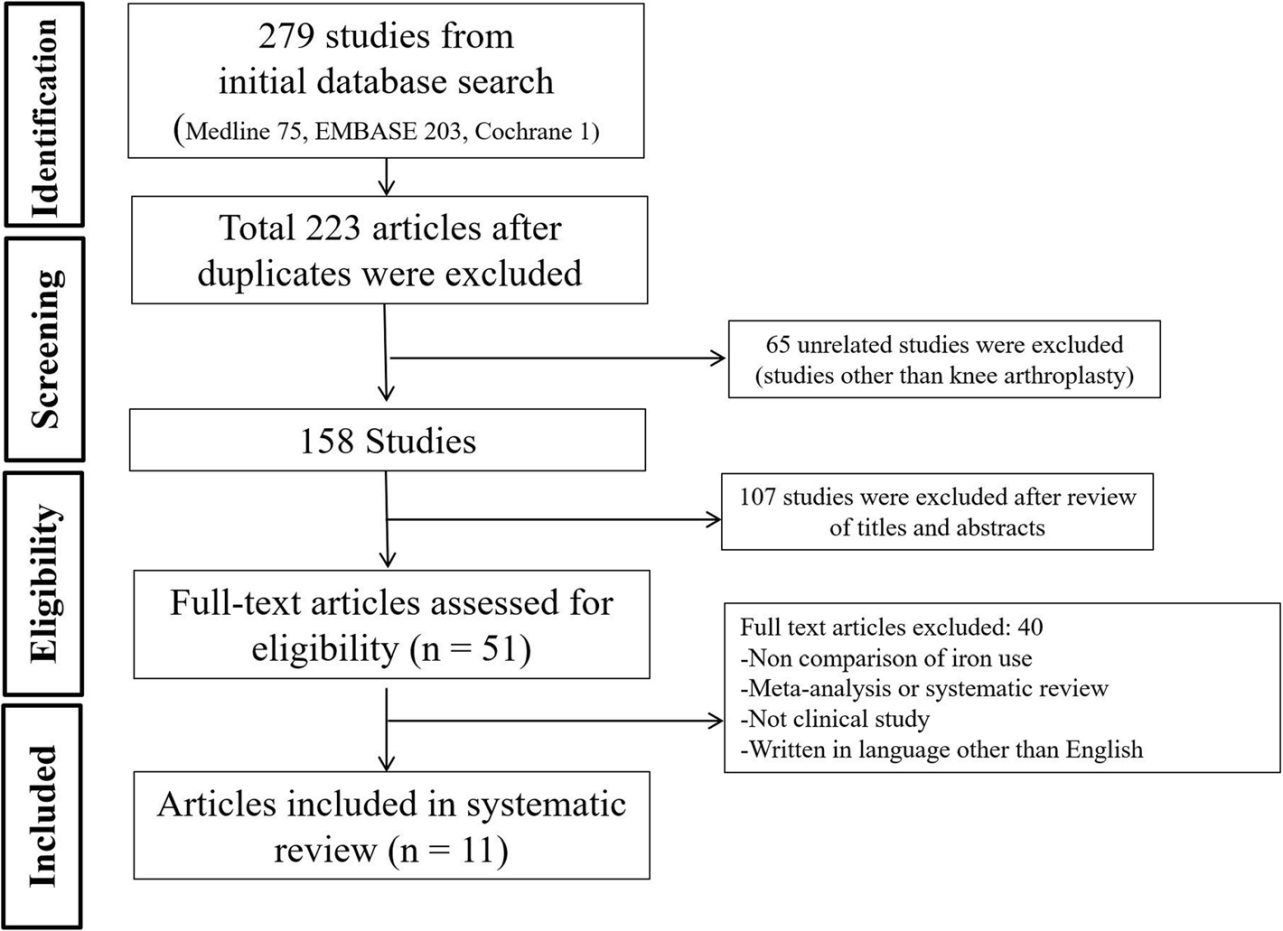
Preferred Reporting Items for Systematic review and Meta-Analysis (PRISMA) flow chart

Eleven articles were included in the final analysis. Seven randomized controlled trials (RCTs) [22–28], one prospective cohort study [29], and three retrospective cohort studies [30–32] were included for the final analysis. Two of them were studies with conflict of interest [23, 32]. The PRISMA flow chart is shown in Fig. 1.

### Quality

All articles underwent quality assessment using the Modified Coleman Criteria [21]. Included studies and modified Coleman Criteria Scoring are presented in Table 1. The studies that we analyzed had an average modified Coleman Criteria Score of 59.5, and eight studies were fair whereas three studies were poor. Most studies compared more than 50 cases and seven studies were RCTs. However, the timing of post-operative hemoglobin measurement was different (1 day to 6 weeks post operation) for each study, and there were differences in indications for allogenic transfusion (hemoglobin < 7 g/dL to < 9 g/dL) as shown in Tables 2 and 3. Quality of the studies was satisfactory.

**Table 1:** Quality of included studies

Author	Journal	Year	Modified Coleman A score	Modified Coleman B score	Modified Coleman Total score
Park et al. [26]	J Clin Med	2019	27	34	61
Heschl et al. [30]	Eur J Anaesthesiol	2018	20	32	52
Biboulet et al. [22]	Anesthesiology	2018	27	37	64
Bisbe et al. [23]	Br J Anaesth	2014	30	37	67
Muñoz et al. [31]	Blood transfus	2014	20	32	52
Muñoz et al. [32]	Br J Anaesth	2012	20	32	52
Na et al. [25]	Transfusion	2011	30	34	64
Gonzalez-Porras et al. [29]	Transfus Med	2009	27	32	59
Mundy et al. [24]	JBJS	2005	27	34	61
Sutton et al. [27]	JBJS	2004	27	34	61
Weatherall et al. [28]	ANZ J Surg	2004	27	34	61

**Table 2:** Evaluation of the effectiveness of iron supplementation by comparing with control group

Author	Year	Journal	Study design	Inclusion criteria	***N*** (iron)	***N*** (control)	OP	Treatment timing	Additional treatment	PBM protocol	Iron	IV or Oral	Dose
Park et al. [26]	2019	J Clin Med	Randomized controlled trial	Hb ≥ 10 g/dLSerum-ferritin < 300 mg/dL (male) or 200 mg/dL (female)	29	29	TKA or THA	Intra-OP			FCM	IV	1000 mg
Heschl et al. [30]	2018	Eur J Anaesthesiol	Retrospective cohort study	Hb < 13 g/dL (men), < 12 g/dL (women)	331	331	TKA or THA	Pre-OP	+EPO		FCM	IV	2*1 g
Biboulet et al. [22]	2018	Anesthesiology	Randomized controlled trial	Hb: 10 ~ 13 g/dL	Oral 50 IV 50		TKA or THA	Pre-OP	+EPO		IV: FCM Oral: Ferrous sulfate	Oral or IV	IV (1000 mg) Oral (160 mg*2/day for 3 weeks)
Bisbe et al. [23]	2014	Br J Anaesth	Randomized controlled trial	Hb: 8.5 ~ 12.0 g/dL	Oral 62 IV 59		TKA	Post-OP		Oral tranexamic acid, tourniquet	IV: FCM Oral: Ferrous sulfate	Oral or IV	IV: single IV injection by Ganzoni formula Oral: 100 mg/day for POD 7 ~ POD 30
Muñoz et al. [31]	2014	Blood transfusion	Retrospective cohort study	Hb ≥ 10 g/dL	182	182	TKA or THA	Post-OP		Tourniquet	Iron sucroseFCM	IV	200 mg (3 consecutive post-operative days) or600 mg FCM on POD 1
Muñoz et al. [32]	2012	Br J Anaesth	Retrospective cohort study	Hb < 10 g/dL on POD 1	Low dose 32High- dose 63	19	TKA or THA	Post-OP			Iron sucroseFCM	IV	100 mg* 3 days 200 mg* 3 days or 600 mg FCM
Na et al. [25]	2011	Transfusion	Randomized controlled trial	Hb ≥ 10 g/dL andserum ferritin < 100 ng/L, orserum ferritin 100 ~ 300 ng/L and transferrin saturation < 20%	54	54	TKA (bilateral)	Intra-OP + Post-OP	+EPO		Iron sucrose	IV	200 mg (Post-OP Hb 7 ~ 8 g/dL, injection 2 times more)
Gonzalez-Porras et al. [29]	2009	Transfus Med	Prospective cohort study	Oral: Hb ≥ 13 g/dL and ferritin < 250 μg/LIV: Hb ≥ 13 g/dL and ferritin < 50 μg/L	80	49	TKA or THA	Pre-OP			Oral: ferrous sulfateIV: iron sucrose	Oral or IV	Oral: 315 mg/day IV: 200 mg/week (minimum 2 weeks)
Mundy et al. [24]	2005	JBJS	Randomized controlled trial	Hb ≥ 13 g/dL (men), ≥ 11.5 g/dL (women)Serum-ferritin < 300 mg/dL (male) or 200 mg/dL (female)	50	49	TKA or THA	Post-OP			Ferrous sulfate	Oral	200 mg three times daily (POD 2 ~ POD 3 weeks)
Sutton et al. [27]	2004	JBJS	Randomized controlled trial	Hb ≥ 12 g/dL (men), ≥ 11 g/dL (women)	35	37	TKA or THA	Post-OP			Ferrous sulfate	Oral	200 mg three times daily (~ 6 weeks after discharge)
Weatherall et al. [28]	2004	ANZ J Surg	Randomized controlled trial	Hb ≥ 12 g/dL	33	34	TKA or THA	Post-OP			Ferrous gluconate	Oral	325 mg (~ 10 weeks after surgery)

*TKA* total knee arthroplasty, *THA* total hip arthroplasty, *Hb* hemoglobin, *EPO* erythropoietin, *OP* operation, *FCM* ferric carboxymaltose, *PBM* perioperative blood management, *IV* intravenous

**Table 3:** Effectiveness of iron supplementation

Author	Year	Journal	Transfusion indication	Transfusion rate_iron (***N***, %)	Transfusion rate_control (***N***, %)	Hb_iron (g/dL)	Hb_control (g/dL)
Park et al. [26]	2019	J Clin Med	Hb < 8 g/dL throughout the perioperative period Hemodynamic instability	2 (6.9%)	4 (12.8%)	Pre-OP (12.5 ± 1.3) POD 1D (10.3 ± 1.4) POD 5D (9.5 ± 1.2) POD 30D (12.8 ± 1.3)	Pre-OP (13.4 ± 1.1) POD 1D (10.7 ± 1.3) POD 5D (9.9 ± 1.6) POD 30D (12.6 ± 1.1)
Heschl et al. [30]	2018	Eur J Anaesthesiol	Hb < 7 g/dL for healthy patients Hb < 8 g/dL for cardiopulmonary restriction patients	40 (12)%	79 (24%)	Pre-OP (12.9 ± 1.7) POD discharge (10.7 ± 1.3)	Pre-OP (12.2 ± 1.1) POD discharge (10.6 ± 1.1)
Muñoz et al. [31]	2014	Blood transfusion	Hb < 8 g/dL throughout the perioperative periodHemodynamic instability	`21 (11.5%)	48 (26.4%)	Pre-OP (13.8 ± 1.2) POD 3D (10.4 ± 1.6) POD 7D (10.6 ± 1.3)	Pre-OP (13.6 ± 1.2) POD 3D (9.2 ± 1.5) POD 7D (9.2 ± 2.2)
Muñoz et al. [32]	2012	Br J Anaesth	Hb < 8 g/dL for healthy patients Hb < 9 g/dL for active cardiac disease	Low-dose: 20 (62%) High -dose: 29 (46%)	16 (84%)	Low-dose Pre-OP (11.8 ± 1.2) POD 1D (8.7 ± 0.8) POD 7D (9.6 ± 0.7) High-dose Pre-OP (12.4 ± 1.3) POD 1D (9.1 ± 0.6) POD 7D (9.8 ± 0.9)	Pre-OP (12.5 ± 1.5) POD 1D (8.7 ± 1.0) POD 7D (10.1 ± 0.7)
Na et al. [25]	2011	Transfusion	Hb: 6 ~ 7: 1 pack 5 ~ 6: 2 pack	11 (20.4%)	29 (53.7%)	Pre-OP (12.1 ± 1.3) Significantly higher Hb level than control group at POD 1D, 2D, 3D, 2 W, 6 W	Pre-OP (12.1 ± 1.2) Significantly lower Hb level than iron supplementation group at POD 1D, 2D, 3D, 2 W, 6 W
Gonzalez-Porras et al. [29]	2009	Transfus Med	Hb < 7 g/dL for healthy patients Hb < 8 g/dL for poor tolerance of anemia Hb < 9 g/dL for cardiac or respiratory failure	Oral: 29 (20%) IV: 10 (20.4%)	96 (31.5%)	Oral Pre-OP (14.3 ± 0.8) POD discharge (10.7 ± 0.1) IV Pre-OP (13.3 ± 0.2) POD discharge (10.3 ± 0.8)	Pre-OP (13.8 ± 1.4) POD discharge (10.7 ± 0.8)
Mundy et al. [24]	2005	JBJS		9 (18%)	5 (10.2)	Men Pre-OP (15.1 ± 1.2) POD 1D (11.0 ± 1.8) POD 5D (10.8 ± 1.6) POD 3 weeks (13.0 ± 1.2) POD 6 weeks (13.8 ± 1.1) Women Pre-OP (13.3 ± 0.9) POD 1D (9.4 ± 1.3) POD 5D (10.3 ± 1.6) POD 3 weeks (11.9 ± 1.1) POD 6 weeks (12.5 ± 0.9)	Men Pre-OP (14.9 ± 0.9) POD 1D (11.4 ± 1.2) POD 5D (10.6 ± 0.9) POD 3 weeks (12.8 ± 0.8) POD 6 weeks (13.3 ± 1.4) Women Pre-OP (13.4 ± 1.3) POD 1D (9.5 ± 1.4) POD 5D (9.8 ± 1.1) POD 3 weeks (11.7 ± 1.4) POD 6 weeks (12.0 ± 1.4)
Sutton et al. [27]	2004	JBJS				Pre-OP (12.7) Post-OP (10.4) At review (12.4)	Pre-OP (12.9) Post-OP (10.5) At review (12.1)
Weatherall et al. [28]	2004	ANZ J Surg				Pre-OP (14.0 ± 1.3) POD 10 weeks (13.3 ± 1.3)	Pre-OP (13.7 ± 1.1) POD 10 weeks (12.8 ± 1.1)

*D* day, *W* week, At review: mean 47 ~ 48 day, *OP* operation, *POD* post-operation day, *Hb* hemoglobin

### Effectiveness of iron supplementation

Effectiveness of iron supplementation was evaluated using nine articles that performed case-control comparison (Table 3) [24–32]. Among them, five articles used intravenously administered iron supplementation [25, 26, 30–32], three used orally administered iron supplementation [24, 27, 28], and one selectively used intravenously or orally administered iron supplementation according to the level of serum ferritin [29]. In each study, the effect of iron supplementation was compared by comparing change in hemoglobin level and transfusion rate after surgery. Among nine studies, one reported significant increase in hemoglobin levels in the iron supplementation group when compared to that seen in the control group, until 6 weeks after the surgery [25]. This study used intravenously administered iron (iron sucrose), along with recombinant human erythropoietin. No significant difference in hemoglobin levels was seen between the iron supplementation group and the control group in the remaining eight studies (Table 3).

**Fig. 2:**
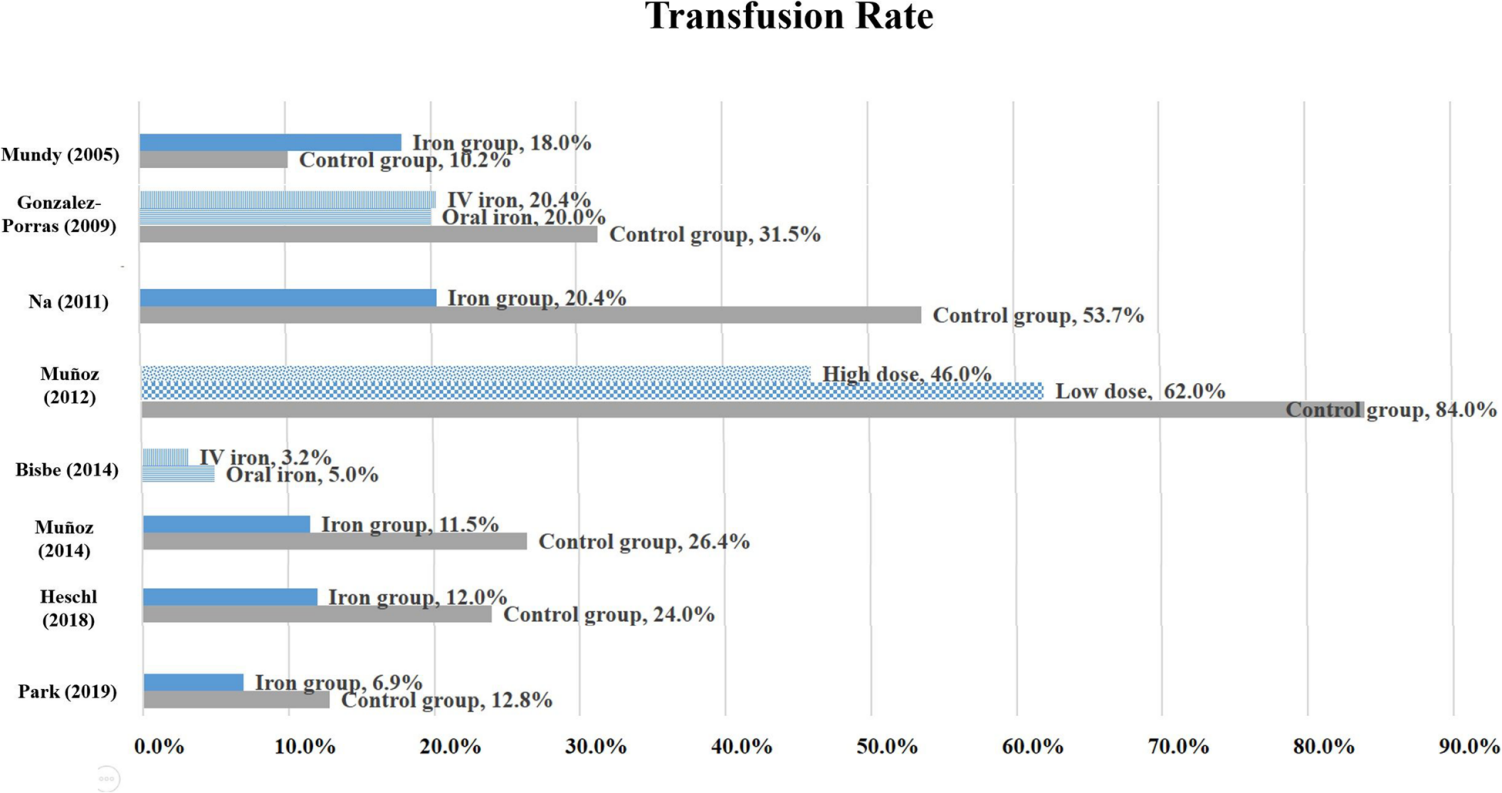
Transfusion rate during the erative period

Seven studies reported transfusion rate which is shown in Fig. 2 [24–26, 29–32]. Figure 2 also include one another study that reported transfusion rates by iron administration method [23]. Among them, two RCTs reported that iron supplementation did not lower transfusion rates, but other five studies reported lowered transfusion rates when compared with those of the control group. Five studies were conducted in patients with anemia (hemoglobin 10 to 13 g/dL or 1-day post-operative hemoglobin < 10 g/dL) [25, 26, 30–32]. One study reported that hemoglobin was higher in the iron supplementation group when compared with that of the control group [25], and four studies reported that iron supplementation was effective in lowering the transfusion rate [25, 30–32]. Another study reported that iron supplementation lowered the transfusion rate and increased iron availability, although the increase was not statistically significant [26].

### Timing of iron supplementation

Iron supplementation was used preoperatively in three studies, post-operatively in six, intraoperatively in one, and used together in one study during intraoperative and post-operative period [25]. Among them, the study that used iron supplementation together in intraoperative and post-operative period reported an increase in hemoglobin levels, when compared with those of control group. Iron supplement was effective in five studies for lowering transfusion rate (two studies that used iron supplementation preoperatively [29, 30], two that used iron supplementation post-operatively and one that used iron supplementation intraoperatively and post-operatively [25]). However, optimal timing of the iron supplement was not evaluated in any of these studies.

### Method of iron administration: orally and intravenously

Three studies compared the efficiency of orally and intravenously administered iron supplementation (Table 4). Two of those studies were RCTs and one was a prospective cohort study [29]. One study reported that intravenous administration of iron supplementation significantly increased hemoglobin levels when compared with those seen in the oral administration of iron supplementation [22], but other studies reported that there was no difference in hemoglobin levels between the two groups, but intravenous administration was more effective in increasing iron availability [23]. In patients with hemoglobin levels < 10 g/dL, intravenous administration significantly increased hemoglobin levels compared to the orally administered supplementation group [23]. Transfusion rates were also compared in two studies, but no differences were reported between the two groups [23, 29].

**Table 4:** Effectiveness of iron supplementation according to the route of administration

Author	Year	Journal	Transfusion indication	Transfusion rate_IV (***N***, %)	Transfusion rate_Oral (***N***, %)	Hb_IV (g/dL)	Hb_Oral (g/dL)
Biboulet et al. [22]	2018	Anesthesiology				Pre-OP (14.9) POD 1D (12.6) POD 3D (12.6) POD 5D (13.4)	Pre-OP (13.9) POD 1D (12.2) POD 3D (11.6) POD 5D (12.3)
Bisbe et al. [23]	2014	Br J Anaesth	Hb < 8 g/dL or acute anemia symptoms	3 (5%)	2 (3.2%)	Pre-OP (13.6 ± 0.9) POD 1D (10.5 ± 1.0) POD 4D (9.7 ± 1.3) POD 30D (11.5 ± 1.2)	Pre-OP (13.6 ± 0.9) POD 1D (10.5 ± 1.0) POD 4D (9.7 ± 1.1) POD 30D (11.0 ± 1.1)
Gonzalez-Porras et al. [29]	2009	Transfus Med	Hb < 7 g/dL for healthy patients Hb < 8 g/dL for poor tolerance of anemiaHb < 9 g/dL for cardiac or respiratory failure	10 (20.4%)	29 (20%)	Pre-OP (13.3 ± 0.2) POD discharge (10.3 ± 0.8)	Pre-OP (14.3 ± 0.8) POD discharge (10.7 ± 0.1)

*D* day, *OP* operation, *POD* post-operation day, *Hb* hemoglobin

### Dose of iron supplementation

Only one study compared the effect of iron supplementation dose [32]. In this study, no difference was seen in the increase in hemoglobin levels corresponding to the iron dose, but the transfusion rate was low in the high-dose iron supplementation group (200 mg iron sucrose for 3 days or 600 mg ferric carboxymaltose) when compared with those seen in the low-dose iron supplementation group (100 mg iron sucrose for 3 days).

## Discussion

This systematic review was performed to answer following questions: (1) Is the iron supplementation necessary during TKA? (2) When is the optimal timing of iron supplementation? (3) Which is better, between orally and intravenously administered iron supplementation? And (4) What is the optimal dose of iron supplementation? Eleven articles were finally included and used for answering these questions. We observed that iron supplementation can help in the treatment of post-operative anemia and lower the transfusion rate, especially in patients who have preoperative anemia. We could not determine the optimal timing for the administration of iron supplementation. Intravenous administration of iron was more effective than oral administration and a high dose of iron was more effective than a lower dose. Most of the included studies stated that iron supplementation has negligible effect on the hemoglobin level, but a confirmative effect on the transfusion rate. However, we believe that it is necessary to consider the time points at which the hemoglobin level was checked.

Recently, a meta-analysis reported the effect of iron supplementation in surgical patients. A meta-analysis by Shin et al. [17] reported that intravenously administered iron supplementation significantly reduces the proportion of transfusion and shortens the length of hospital stay in orthopedic surgery. However, a meta-analysis by Koo et al. [18] reported that while intravenously administered iron supplementation may increase the post-operative hemoglobin level, transfusion rates cannot be reduced in surgical patients. A meta-analysis by Yang et al. [19] reported that iron supplementation raises the hemoglobin level but does not affect the transfusion rate. However, these studies were performed in different conditions and it is difficult to compare blood loss directly due to the differences in the amount of bleeding. Additionally, indications of the transfusion could also be different, and even for the same operation, the amount of blood loss varies according to the surgical method and technique of the surgeon. Therefore, only comparative studies such as ours can assess the efficacy of the iron supplementation.

Intravenously administered iron supplementation was a similar or superior method for increasing hemoglobin levels and lowering transfusion rate compared to orally administered iron supplementation. However, a high intravenously administered iron dose (ferric carboxymaltose) was used in two studies [22, 23], and a low intravenously administered iron dose was used (iron sucrose) in one study [29], so it is thought that there was an effect according to the administration dose as well as the administration method. A meta-analysis by Koo et al. [18] reported that intravenously administered iron was better for increasing hemoglobin levels than orally administered iron, but both methods affected the transfusion rate in a similar manner. As very few studies compared the timing of iron supplementation, we could not determine the optimum timing for the administration of iron supplementation. However, as it is widely believed that hemoglobin level rises in 2 to 4 weeks after iron supplementation, surgeons should consider this for determining the optimum timing for the administration iron supplementation [33]. A meta-analysis by Shin et al. [17] reported that iron dose does not affect the transfusion rate. In our analysis, a high dose of iron lowered the transfusion rate. As there was only one study on iron dose, we believe that this could be insufficient evidence to draw any conclusion about the iron dose.

Iron supplementation often translates into additional costs for patients. Considering the increase in length of stay and the cost of transfusion, iron supplementation could be cost-effective, although direct comparison has not been performed [31, 34]. However, the cost can differ according to each country’s health care system and insurance status, and it may not be available in some countries. It has also been reported that the use of iron supplementation can reduce the length of hospital stay [35, 36]. Therefore, further studies are necessary to evaluate the effects and possible complications of iron supplementation when compared with the transfusion, and determine its optimal form, dose, and timing. Moreover, we believe that RCTs that are identical in terms of the type, dose, and timing of the iron supplementation are necessary. Additionally, studies that accurately measure blood loss, hemoglobin levels, ferritin levels and its saturation rate at regular time intervals are also necessary.

The present study also has some limitations. First, only one of the included studies was conducted with unilateral TKA alone, whereas the rest of the studies included bilateral TKA or THA. This may have influenced the results of our study due to the difference in the amount of blood that was lost. Second, iron type, dose, timing, and indication of iron supplementation were not identical in all the studies, and the time point of hemoglobin-level estimation was also different. Therefore, we decided to perform systematic review than meta-analysis. Third, the publication years of the included studies were also different. With the current advancement of iron formulations, recent studies have shown favorable results for iron supplementation, with a distinct possibility of improvement in future. Fourth, in some studies, other formulations were also used along with iron supplementation for reducing blood loss, such as tranexamic acid and erythropoietin.

## Conclusion

Iron supplementation is not clear as a way to raise hemoglobin after TKA, but an effective treatment for lowering transfusion rate, especially in patients with preoperative anemia. We could not determine the optimal timing and dose of the iron. Intravenously administered iron was similar to, or better than, orally administered iron for improving hemoglobin levels and transfusion rates.

## Data Availability

All data generated or analyzed during this study are included in this published article.

## Abbreviations

TKA: Total knee arthroplasty
THA: Total hip arthroplasty
TJA: Total joint arthroplasty
